# A Decision Support System for Drinking Water Production Integrating Health Risks Assessment

**DOI:** 10.3390/ijerph110707354

**Published:** 2014-07-18

**Authors:** Ianis Delpla, Donald T. Monteith, Chris Freeman, Joris Haftka, Joop Hermens, Timothy G. Jones, Estelle Baurès, Aude-Valérie Jung, Olivier Thomas

**Affiliations:** 1 Advanced School of Public Health, Sorbonne Paris Cité, Avenue du Professeur Léon Bernard, Rennes Cedex CS 74312, France; E-Mails: ianis.delpla@crad.ulaval.ca (I.D.); estelle.baures@ehesp.fr (E.B.); audevaleriejung@ecole-eme.fr (A.-V.J.); 2 Inserm, U 1085-Institute of Research in Environmental and Occupational Health (IRSET), LERES, Rennes 1 University, Avenue du Professeur Léon Bernard, Rennes Cedex CS 74312, France; 3 European University of Brittany, Boulevard Laënnec, Rennes 35000, France; 4 École Supérieure d’aménagement du territoire et de Développement Régional, Laval University, Pavillon F.A. Savard, Ste-Foy, QC G1K 7P4, Canada; 5 Centre for Ecology and Hydrology, Lancaster University, Lancaster LA1 4AP, UK; E-Mail: donm@ceh.ac.uk; 6 Wolfson Carbon Capture Laboratory, School of Biological Sciences, Bangor University, Deiniol Road, Bangor, Gwynedd LL57 2UW, UK; E-Mails: c.freeman@bangor.ac.uk (C.F.); t.jones@bangor.ac.uk (T.G.J.); 7 Toxicology Division, Institute for Risk Assessment Sciences, Utrecht University, Utrecht 3508 TD, The Netherlands; E-Mails: j.haftka@uu.nl (J.H.); j.hermens@uu.nl (J.H.); 8 School of Environmental Engineering, Campus de Ker Lann, Avenue Robert Schumann, Bruz 35170, France

**Keywords:** decision support system, drinking water, small and medium scale water services, health risks assessment, climate change, organic carbon

## Abstract

The issue of drinking water quality compliance in small and medium scale water services is of paramount importance in relation to the 98/83/CE European Drinking Water Directive (DWD). Additionally, concerns are being expressed over the implementation of the DWD with respect to possible impacts on water quality from forecast changes in European climate with global warming and further anticipated reductions in north European acid emissions. Consequently, we have developed a decision support system (DSS) named ARTEM-WQ (AwaReness Tool for the Evaluation and Mitigation of drinking Water Quality issues resulting from environmental changes) to support decision making by small and medium plant operators and other water stakeholders. ARTEM-WQ is based on a sequential risk analysis approach that includes consideration of catchment characteristics, climatic conditions and treatment operations. It provides a holistic evaluation of the water system, while also assessing human health risks of organic contaminants potentially present in treated waters (steroids, pharmaceuticals, pesticides, bisphenol-a, polychlorobiphenyls, polycyclic aromatic hydrocarbons, petrochemical hydrocarbons and disinfection by-products; n = 109). Moreover, the system provides recommendations for improvement while supporting decision making in its widest context. The tool has been tested on various European catchments and shows a promising potential to inform water managers of risks and appropriate mitigative actions. Further improvements should include toxicological knowledge advancement, environmental background pollutant concentrations and the assessment of the impact of distribution systems on water quality variation.

## 1. Introduction

Drinking water systems supplied by surface waters are vulnerable to short-term variation in inputs of organic matter that affect raw water quality. Increases in rainfall intensity and the frequency of heavy rainfall events and droughts are predicted for the end of the century [1]. Such changes could strongly affect water quality and treatment operations [1]. Rainfall events, for example, can cause rapid degradation of water quality, including elevated levels of Total Suspended Solids, Dissolved Organic Carbon (DOC) and Total Organic Carbon (TOC), nutrients, some micropollutants with high partition coefficients, and microbiological parameters such as bacteria, viruses or protozoa [2]. Conversely, drought events and their aftermath can lead to algal blooms and associated increases in cyanotoxins, sediment micropollutants release, diminution of dissolved oxygen, and of the river dilution capacity for nutrients (ammonium, orthophosphates) or heavy metals [3,4,5,6]. To complicate matters further, DOC concentrations have been rising steadily across many areas of northern and central Europe and North America in recent decades, primarily in response to the influence of declining acid deposition on organic matter solubility [7,8,9,10,11], and are of great concern for drinking water producers. Changes in land-use practice (e.g., [12]) and climate [7,13,14,15] have also been identified as potential drivers of DOC increases.

Future hydrological changes coupled with a long-term rise in organic matter solubility may therefore increase loads of organic matter and associated micropollutants in “raw water”, impairing water treatment efficiency and leading to a rise in disinfection by-products [16]. Large water supplies have generally robust and adaptive water treatment processes but there is a concern about the adaptability of small water supplies.

Small and medium scale water services supplying water to fewer than 10,000 inhabitants are commonplace in Europe and supply almost one third of the population, especially located in rural areas [17,18]. In Europe, it was shown that more than one third of the small water supply systems (SSWS) delivered water not complying with the values set in the 98/83/CE European Drinking Water Directive (DWD) [19]. Technical and management difficulties, combined with a lack of financial resources were identified as the main limitations to ensuring good water quality distributed by SSWS [19,20]. Moreover, small supplies are often located in isolated areas, and operators lacking easy access to expert assistance were shown to be highly vulnerable to sudden changes in raw water quality.

The DWD revision process, started in 2003, was concluded in 2011 with the statements that no revision of legislation was required but that increased implementation and enforcement efforts using a risk-based approach would be necessary to ensure safe drinking water in smaller supplies [19]. In this context, decision support systems (DSS) have the potential to provide valuable tools to enable improved and informed management of SSWSs.

DSSs are defined as any system supporting decision making and include executive information systems, executive support systems, geographic information systems, and on-line analytical processing and software agents, and are normally underpinned by computer-based models [21]. During the last few decades, many DSSs have been developed to inform water resource management issues such as prevention of water shortages (droughts), surpluses (floods), and water quality impairment (pollution) [22]. Some DSSs have been designed to inform management of large rivers basins such as Elbe [23,24], or big cities [25], while others have focused on tackling issues inherent to the high degree of uncertainty existing in water systems due to their complexity [26,27,28].

Water quality-based DSSs are increasingly being developed to underpin the management and protection of water resources under the Water Framework Directive [22,29,30,31,32,33,34]. Less attention has been given to DSS development with respect to informing implementation of the DWD although there is a clear need to establish holistic approaches in this field linking resources to the consumer. However, some decision support tools and technical guides, principally based on a semi-quantitative approach (Table 1), have recently been developed to assist managers of SSWSs in the assessment of risks to their water systems [26,35,36,37,38,39].

These various tools or guides are mainly based on the water safety plan (WSP) approach defined by the World Health Organization (WHO) and adopted worldwide. In its third edition of the guidelines for drinking water quality, the WHO also concluded that a comprehensive risk management approach is the most effective way of ensuring the safety of drinking water supplies [40]. The WSP approach includes hazard ranking for qualitative (or semi-quantitative) risk assessments (RA). This is especially relevant for small supplies, as the frequency of monitoring is usually low. Due to the potentially threat to health posed by waterborne disease following the spread of pathogens within a distribution system, and concerns over the ability to detect such outbreaks, these tools are mainly focused on microbiological risk assessment and management. However, chemical risks posed by cyanotoxins, nitrates and disinfection by-products may also be high. Moreover, new issues related to micropollutants, emerging substances or environmental changes are not taken into account by these tools/guides. Finally, the impact of further anticipated changes in DOC loads and concentrations on the viability of a water treatment system need to be factored in. As concerns about health impacts related to water consumption become increasingly societally important, there is a clear need for a management tool capable of integrating this information.

**Table 1:** Summary of the decision support systems (DSS) designed for studies of small water supply systems (SSWS).

Leading Organization	Year	Name of The Tool	Main Characteristics	Water Supply Size Range	Application Environment	Outputs
WHO/IWA	2009	Water Safety Plan Manual	Guidance document	2500–8,500,000	Agricultural (extensive) Urban	Risk scoring Control measures
Ireland (EPA)	2010	Handbook on implementation for Water Services Authorities for private water supplies—Section 10	Semi quantitative RA	2–5000	Agricultural Urban pressure	Risk scoring
United Kingdom (Scottish executive)	2003	Private water supplies : Technical Manual	Semi quantitative RA	1–50 50–1000	All pressures (wild life, agriculture, forestry industry, wastewater, sludge, landfill	Risk scoring Recommendations
France (ASTEE)	2009	Ogeris, aide à l’évaluation des risques microbiologiques dans les petites unités de production/distribution d’eau potable	Vulnerability assessment	<5000	All types	Recommendations and priorization of actions
Germany (DVGW)	2008	Technical note for guideline W 1001. DVGW Rules, security of water supply risk management during normal operation	Guidance document			Risk scoring Control measures
USA (EPA)	2007	HACCP Strategies for Distribution System Monitoring, Hazard Assessment and Control	Guidance document	Large supplies (1 example 770,000)	Urban pressure	Risk analysis and recommendations
University of Guelph (Canada)	2009	Fuzzy-Logic Modeling of Risk Assessment	Fuzzy logic and fault tree methodology	Small water supplies	All types	Identification of which failure contributes to the high potential risk Recommendations

The ARTEM-WQ decision support system (AwaReness Tool for the Evaluation and Mitigation of drinking Water Quality issues resulting from environmental changes) has been designed in light of DWD requirements for SSWS, forecast changes in the European climate in response to global warming and further anticipated reductions in north European acid emissions and resulting DOC changes. Its main purpose is to provide water stakeholders (sanitary authorities, water treatment operators and other stakeholders) with a holistic tool for identifying and assessing the risks posed by the complex range of pressures (agricultural, industrial, climatic, *etc.*) on water resources. Moreover, the tool should enable operators to specify plant characteristics in a consistent manner, and inform on appropriate mitigation measures for enabling improvement of drinking water safety in small and medium supplies. Finally, the tool is designed to assess the viability of a water treatment system in the context of further anticipated changes in DOC loads and concentrations.

ARTEM-WQ is targeted primarily at water system managers and operators of small and medium scale treatment plants, and particularly those responsible for plant construction or renovation. However, it should also be of value in informing sanitary authorities responsible for sanitary quality monitoring and river basin managers (e.g., Agence de l’eau, DDTM, ONEMA, DREAL).

## 2. Description of the Tool

### 2.1. General Architecture

The general architecture takes the following sequential approach:

1. Water resources risk analysis based on a score determined from data on catchment land-use and land management.
2. Water treatment risk analysis.
3. Risk reduction recommendations to support decision making.
4. Health risk assessment.

From the outset, it was necessary to distinguish between systems dominated by waters draining unimproved upland environments where riverine organic matter is chiefly derived from terrestrial unfertilized primary production, and more anthropogenically impacted catchments, *i.e.*, those draining land supporting improved or intensive agriculture, urban settlement and industry.

Vulnerability is defined as the degree to which a system is susceptible to, and unable to cope with, adverse effects of a hazard, such as climate change [1]. The tool requires completion of a sequence of five mandatory questionnaires that together describe drinking water production system vulnerability. The questionnaires are based primarily on hazard parameters derived from a review of existing guidance documents used for risk assessment and risk management of water supplies (*cf.* Table 1). These have been discussed and refined through meetings with project partners and local stakeholders. 

A semi-quantitative risk scoring approach is defined as a combination of the likelihood of a hazard and the severity of its consequences [38,39]. This approach is used to assess the overall risks resulting from the presence and relative magnitude of the listed hazards, with higher scores indicating greater potential severity of consequences for the safety of water supply. This allows identification and assessment of all risks in the catchment and the treatment plant that may result in a risk to health and/or a breach of the required standard [39]. Risks are accorded positive scores while risk reducing measures such as treatment management actions or water quality improvement actions plans are treated as negative.

Moreover, the DSS includes the capacity to predict DOC concentration for unimproved upland catchments. The future target date chosen is the medium term 2050 horizon. This DOC model may be used to predict average DOC concentration in unimproved upland catchments that are largely free of soil erosion after completion of “Natural catchment” and “Treatment” questionnaires if the user determines that the catchment is subject to only slight human impact (by selecting the appropriate box in the “catchment type” tab).

Others tool components include:

1. Graphical representation of scores.
2. Recommendations for catchment and treatment management improvement.
3. Estimates of likely pollutant concentration following treatment and associated Health Risk Assessment (HRA).

**Figure 1:**
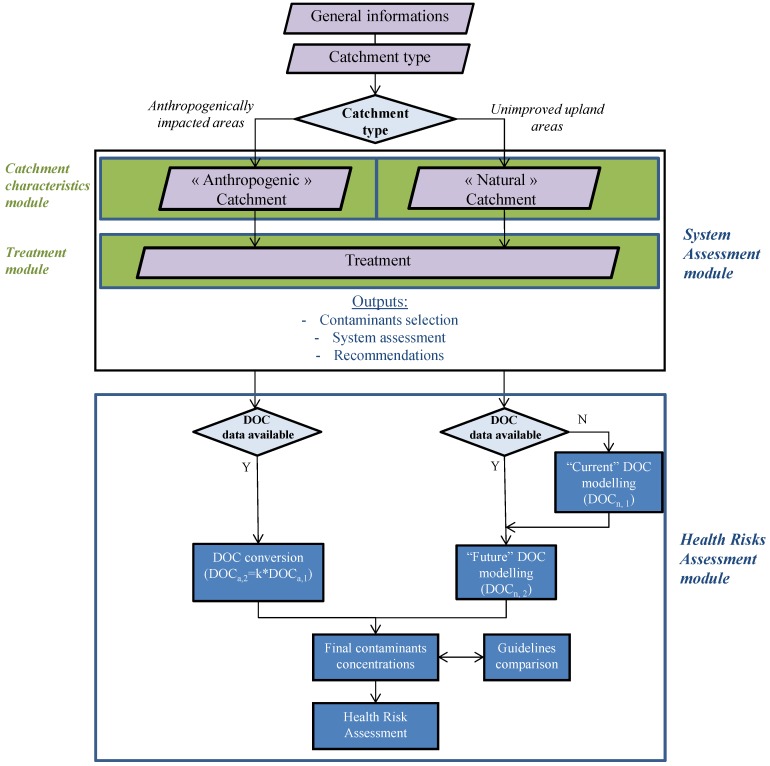
General architecture of the ARTEM-WQ Decision Support System.

The HRA focuses on potentially hazardous substances predicted from the pressures identified for the catchment in question, the potential influence of raw water quality by considering DOC concentration, and the impact of treatment on substances removal and disinfection by-products formation. Chemical contaminants are generally present in very low concentrations in drinking water resources. The effects of chronic exposure to small quantities of contaminants in drinking water have been the most studied endpoint these last decades and lead to a consequent production of toxicological literature [41] that is useable for conducting a human health risk assessment. Consequently, we decided to conduct the HRA for carcinogenic (“non-threshold”) effects following ingestion.

The DSS architecture is detailed in Figure 1.

### 2.2. Parameters

Dissolved Organic Carbon, the primary focus of this DSS, is a key water quality parameter for drinking water production as its concentration and quality influence micropollutants adsorption, Disinfection By-Product (DBP) formation and biological regrowth within the distribution system. High levels of organic matter in raw waters sources are known to reduce clarifier performance, and can also significantly increase drinking water treatment costs due to a higher chlorine and coagulant demand [16].

Overall, a total of 109 organic contaminants were considered for inclusion in the DSS. We included several frequently detected micropollutants of surface waters such as steroids (estradiol, estrone), pharmaceuticals (carbamazepine, diazepam and triclosan), pesticides (e.g., atrazine, bromophos methyl, cypermethrin, deltamethrin, permethrin, aldrin, dieldrin, DDD, DDE, HCH), bisphenol-a, polychlorobiphenyls (e.g., PCB 77, 118, 126, 156, 169, 170, 180), polycyclic aromatic hydrocarbons (e.g., phenanthrene, pyrene, benz(a)anthracene) and petrochemical hydrocarbons (e.g., 7,12-dimethylbenz(a)anthracene, nonane). Disinfection by-products such as the four THM species (chloroform, bromoform, dichlorobromomethane and chlorodibromomethane) and two haloacetic acids (dichloroacetate, trichloroacetate) were also included.

Where no DOC measurements were available for waters draining unimproved upland habitats that are not subject to significant soil erosion, the DSS allows estimation of average concentrations using a simple statistical model based on the relationship between long-term mean DOC concentration and physico-geographic data representing mean catchment altitude, percentage peatland extent, and effective precipitation (*i.e.*, precipitation minus evaporation) according to [42].

### 2.3. Main Functionalities

#### 2.3.1. Catchment Type Module

This questionnaire is used to classify the river basin according to dominant environmental pressures into either anthropogenically impacted or anthropogenic and “natural”; on the basis of land use. This simple river basin characterization system represents the first step in the tool, directing the user toward further questionnaires.

#### 2.3.2. Catchment Characteristics Module

Completion of this questionnaire generates an assessment of drinking water catchment area vulnerability. The questionnaire content was adapted from [35]. It requires data on the type of land use, animal density, agricultural practices in the catchment (farm waste spreading/storage, *etc.*), urban and industrial pressures (industries, hospitals, tourism infrastructures, WWTP, landfill sites, storm overflows, *etc.*), catchment characteristics (slopes) and management measures. The questionnaire’s contents are generally similar for both major catchment types, but with additional data required for the impacted systems. A description of the considered catchment characteristics with their corresponding scores is given in Supplementary Information.

#### 2.3.3. Treatment Module

Here, the efficiency of the drinking water treatment plant (DWTP) system is evaluated on the basis of data on monitoring measures, treatment performance and routine plant exploitation (inspections, control manuals, bypass). The questionnaire content was adapted from [35]. A total of fifteen of the most commonly applied treatments steps are included, e.g., sand filtration, coagulation, activated carbon (granular/powder), membrane filtration, disinfection (chlorination, UV, ozonation). In this questionnaire, treatment steps and performance, appropriate monitoring measures, and adequate plant management are considered as risk-reducing measures and therefore generate negative scores. A description of the treatment questionnaire with its corresponding scores is given in Supplementary Information (Tables S1–S3).

#### 2.3.4. System Assessment Module

**Figure 2:**
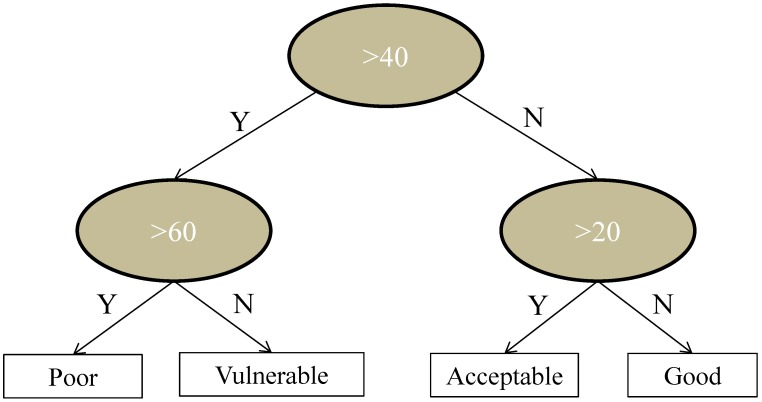
Water quality assessment tree used in ARTEM-WQ showing the three different system assessment scores’ thresholds (20, 40 and 60).

Scores generated by the “Catchment characteristics” and “Treatment” questionnaires are summed, as it was commonly done in other DSS [35,36]. Firstly, the net score is graphically presented, associated with a recommendation according to the net score value. The following algorithm is used for water quality assessment (Figure 2).

The thresholds were defined following a sensitivity analysis of the sum of risk scoring provided by the DSS by testing the tool in two extremes situations (pristine catchments with adequate water treatment and management; substantially polluted catchments with insufficient water treatment and management). The scores range from 0–94. Data for a considerably wider range of sites will be necessary to provide a robust calibration of the risk score (see Discussion), but the example in Figure 2 is based on the assumption that it is proportional to the risk of delivering an unsafe drinking water to the consumer. Consequently, in this example the score range has been divided equally into four water quality classes (0–20, 21–40, 41–60 and >60).

**Figure 3:**
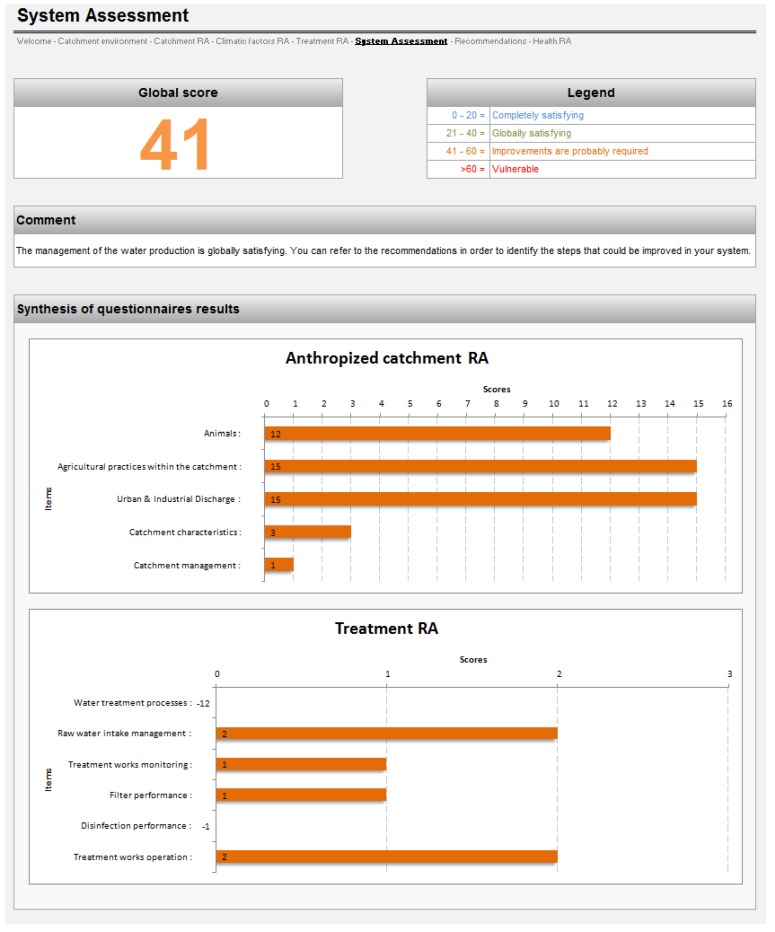
Screenshot of the System Assessment tab.

An example of the System Assessment output is provided in Figure 3. The bars in the charts convey scores for chief sub-categories determined by the Catchment and Treatment questionnaires. Positives scores are marked in orange, while scores of zero or below are provided numerically. This presentation allows the user to rapidly assess the key potential pressures and a range of water treatment operations that might be improved (Figure 3).

**Figure 4:**
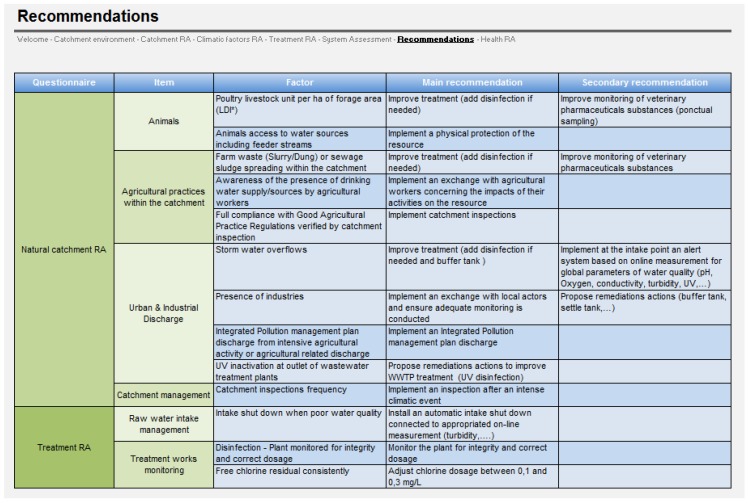
Screenshot of the Recommendations tab.

A corrective action is proposed for each potential hazard identified on the water system (*i.e.*, for positive risk scores), such as high animal density, discharges from wastewater treatment plants, or inadequate water treatment plant management. Corrective actions cover the following fields: river basin management, water treatment process design, maintenance, performance and monitoring. These corrective measures include on one hand primary recommendations which are strongly suggested to be put in place, and on the other hand secondary recommendations which could be put in place further or are not mandatory by the regulation. For instance, if there is evidence of storm water overflows previously occurring in the catchment, the primary recommendation given by the software is to implement a buffer tank and the secondary recommendation is to implement at the intake point an alert system based on online measurement for global parameters of water quality (Figure 4).

#### 2.3.5. Health Risk Assessment Module

The objective of this module is to highlight the substances that could present a health risk. Contaminant concentrations are calculated and a Health Risk Assessment is performed for each substance. The module requires input data on DOC concentrations that are derived either directly from measurements or by modeling. Then, the calculation of organic contaminants’ removal following water treatment is achieved. Finally, the final concentrations obtained are used as inputs to calculate individual estimates of risks for each substance following an exposure by ingestion.

##### 2.3.5.1. Contaminant Selection

Potentially harmful contaminants are selected according to the pressures identified on the river basin in the “Catchment” questionnaire. Each pressure is linked with one or more groups of substances (Ex: PAH substances with “Presence of industries”). If the score associated with one pressure is strictly positive, all substances potentially present and associated to this pressure are taken into account.

##### 2.3.5.2. Dissolved Organic Carbon Concentration Calculation

This module is intended to allow the estimation of contemporary DOC concentration in unimproved upland areas where DOC data are absent, in addition to providing estimates of likely future levels on the basis of IPCC climate change scenarios. 

In the case of using the DSS for unimproved upland environments (“Natural catchments” in Figure 1) and when DOC data are not available, a dialog box allows the estimation of DOC concentrations from geo-physical data (DOC model). The current DOC model provides an estimate of contemporary DOC concentrations (*i.e.*, at the time of the DSS use) but is also being developed to predict long term trends according to current climate change scenarios (2050) in natural environments in the UK region. Input variables are based on parameters identified as dominating spatial variation in DOC concentrations variations in UK upland catchments according to [42].

1. Mean catchment altitude.
2. Percentage of peat or peaty gley soil in the catchment.
3. Annual effective rainfall (*i.e.*, rain minus evaporation).

When using the DSS for directly anthropogenically impacted environments (“Anthropogenically impacted catchment” in Figure 1), the user needs to provide DOC data for pursuing the Health Risk Assessment. In order to provide a potential future scenario DOC concentration, we assumed a worst case scenario stating a 50% increase in contemporary levels, on the assumption that trends experienced in Brittany [43] over the last 30 years will continue into the future at the current rate. Consequently, for long term trends’ concentrations (2050), the following equation was used: DOC_a,2_ = k × DOC_a,1_, (with k = 1.5, DOC_a,1_: DOCconcentration provided by the user).

##### 2.3.5.3. Calculation of Organic Contaminants Removal Following Water Treatment

In order to remove dissolved (and colloidal) organic carbon (DOC), coagulation and subsequent flocculation precedes most water treatment operations. Hydrophobic organic contaminants sorbed to DOC will also be removed from solution by coagulation/flocculation processes [44]. Organic chemicals that are strongly sorbed to the hydrophobic fraction of DOC will therefore be more efficiently removed by coagulation/flocculation compared to hydrophilic compounds that are weakly sorbed to DOC.The removal efficiency of organic contaminants will also be dependent on the hydrophobicity of the compound with higher removal efficiencies observed for chemicals with higher hydrophobicity [45]. It is assumed in this work that the organic contaminant is solely sorbed to the hydrophobic fraction of DOC and there is no appreciable sorption of freely dissolved chemicals to the precipitated flocs or to the residual inorganic coagulants.

The removal of an organic contaminant sorbed to DOC was subsequently calculated with the following equation:

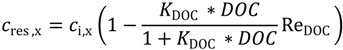
(1)
where *c*_res,x_ is the residual contaminant concentration after coagulation of compound x, *c*_i,x_ is the initial contaminant concentration in the raw water, *K*_DOC_ is the DOC to water partition coefficient, DOC is the DOC concentration of raw water and Re_DOC_ is the removal efficiency of DOC following coagulation.

Other water treatment steps can subsequently remove the residual contaminant concentration after the coagulation/flocculation process. Water treatment can include e.g., sand filtration, (pre-)disinfection with either UV/H_2_O_2_, O_3_, Cl_2_ or NaClO_2_, filtration with granular activated carbon and finally (post-) disinfection. Different removal efficiencies (e.g., for treatment a and b following coagulation/flocculation) are combined, according to the following equation:

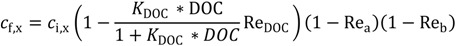
(3)
where *c*_f,x_ is the final contaminant concentration in finished drinking water, and Re_a,b_ is the removal efficiency of a specific organic contaminant and water treatment.

**Figure 5:**
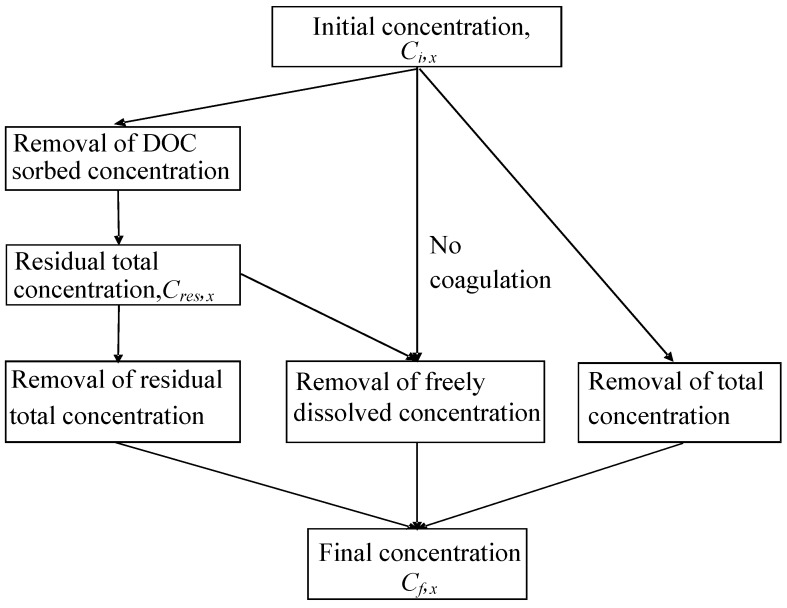
Influence of Dissolved Organic Carbon on the calculation of initial and final concentrations during preparation of drinking water via removal of (1) DOC sorbed and residual contaminant concentration; (2) freely dissolved contaminant concentration; and (3) total contaminant concentration.

The removal of an organic contaminant following a sequence of different water treatment processes is schematically shown in Figure 5, where the removal via coagulation and any other water treatment is shown in the left of the figure.

In case there is no coagulation/flocculation step at a water treatment plant, organic contaminants may also be removed via the freely dissolved or the total concentration, respectively (Figure 5). The final concentration after removal of the freely dissolved concentration from solution can theoretically be calculated but there is no literature data available on the removal efficiencies of freely dissolved contaminants. For this reason, removal of freely dissolved concentration was not considered here.

This step has required an extensive dataset constitution for grouping relevant data on environmental background chemicals concentrations, organic contaminant sorption to DOC (*K*_DOC_) and treatment efficiency (removal rates). *K*_DOC_ values were obtained following sorption experiments conducted by the Utrecht University (data not shown). Data on background concentrations and removal rates by treatment was obtained following a large literature review [46,47,48,49,50,51,52,53,54,55,56,57,58,59,60,61,62,63,64].

When a group of substances is suspected to be found in raw waters, the DSS synthesize all initial concentration values (C_i,x_), corresponding values of *K*_DOC_ (*K*_DOC,x_), DOC concentration in treated waters (*DOC*_theo2_, theoretical or measured, after validation of “Treatment” questionnaire) and the removal coefficient(s) corresponding to the water treatment(s) selected by the user (“Treatment” questionnaire), in order to calculate final concentration values (after treatment).

##### 2.3.5.4. Calculation of Disinfection By-Product Concentrations

The calculation of DBPs concentration (4 THM, 2 HAA) is realized by compound-specific equations:

For THM, the following equation is used [65]:

TTHM = 1.392(DOC)^1.092^ (pH)^0.531^ (T)^0.255^ (3)

where TTHM = Total Trihalomethanes (µg/L), DOC = Dissolved Organic Carbon concentration (mg/L), T = Mean Water Temperature at Montfort DWTP (°C) = 12.8 °C, pH = Mean pH value at Montfort DWTP = 7.8.

A partition coefficient is also applied for the calculation of each THM substance. These coefficients are taken from the results of the PollProx study (219 samples in Rennes area) [66].

In this survey, the four THM are distributed as follows:

Chloroform: 23.5%
Bromoform: 28.2%
Dibromochloromethane: 30.2%
Bromodichloromethane: 18.4%

Hence, the formula becomes:

Chloroform = 1.6 × (DOCtreated water)^1.092^
Bromoform = 1.92 × (DOCtreated water)^1.092^
Dibromochloromethane = 2.057 × (DOCtreated water)^1.092^
Bromodichloromethane = 1.253 × (DOCtreated water)^1.092^

For the HAA calculation, the following formula is used [67]:

Dichloroacetate = 1.534 + 0.566 × (Bromodichloromethane) − 0.258 × (Bromoform)
Trichloroacetate = 1.269 + 0.375 × (Chloroform)

##### 2.3.5.5. Individual Excess Risks Calculation (Non-Thresholds Effects)

The last step involves the calculation of Individual Excess Risks (IER) for each substance for carcinogenic (“non-threshold”) effects for an adult following a lifetime exposure by ingestion. Since the selected substances are very different in structure and toxicity, the quantitative health risk assessment is performed for each one separately. Relevant toxicological information was collected by consulting ten different toxicological databases (Integrated Risk Information System-USEPA, Agency for Toxic Substances and Disease Registry, Health Canada, International Toxicity Estimates for Risk-TERA, Office of Environmental Health Hazard Assessment, Dutch National Institute for Public Health and the Environment-RIVM, Risk Assessment Information System, Hazard Substances DataBase, Ineris and Furetox-InVS).

The following equation, derived from [68], is used for the IER calculation:

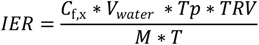
(4)
where C_f,x _= Substance final concentration (THM, other, …) (mg/L), TRV = Toxicological Reference Value (mg/kg/d)^−1^, Vwater = Daily water consumption, set to 2 L/day, M = Mean adult body weight, set to 70 kg, T = Lifetime, set to 70 years, Tp = Exposure duration (ingestion), set to 70 years. A mean exposure to 2 L/day of water during the lifetime (70 years) was considered.

**Figure 6:**
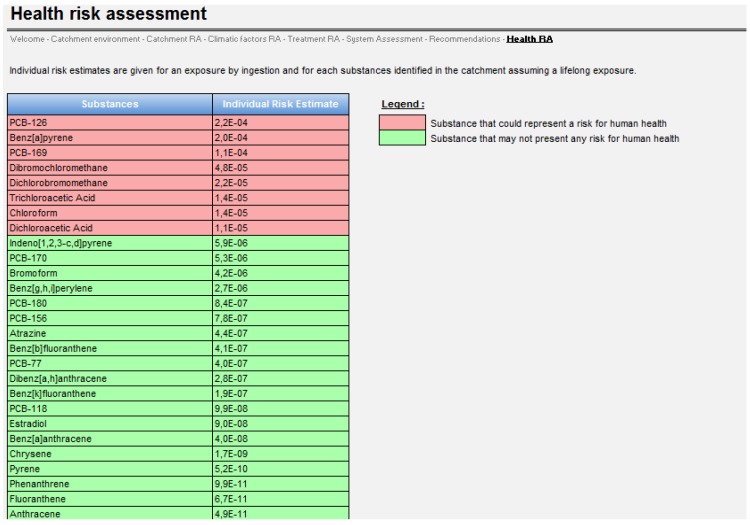
Screenshot of the Health Risk Assessment tab.

Results of the quantitative health risk assessment are presented for individual substances. The software highlights within a new tab risk values higher than 10^−5^ in red and the values lower than 10^−5^ in green. This threshold corresponds to the guideline set by WHO for defining risk as “acceptable” [69]. These substances are ordered in IER descending order.

A screenshot of the Health Risk Assessment tab is presented in Figure 6.

## 3. DSS Validation

The methodology used in the DSS was validated by applying it to eight water systems located in Brittany (France). Sites were selected to span a range of key pressures and on the basis of available data. The systems covered a wide range of river basins environments ranging from natural forested ecosystems to highly intensive agricultural zones and urbanized catchment. Among the catchments, six are used as a water resource to produce drinking water.

The proportion of agricultural land varies from 16.1% for the less human-impacted catchment to 96.1% for the site with the most intensive agricultural practices. The water treatments are relatively complex for all the sites and all include different steps such as coagulation, chlorination, ozonation and granular activated carbon filtration and/or sand filtration. Data on water treatments was obtained for five sites, corresponding to the most anthropized environments.

Questionnaires scoring and substances selection were validated separately.

**Figure 7:**
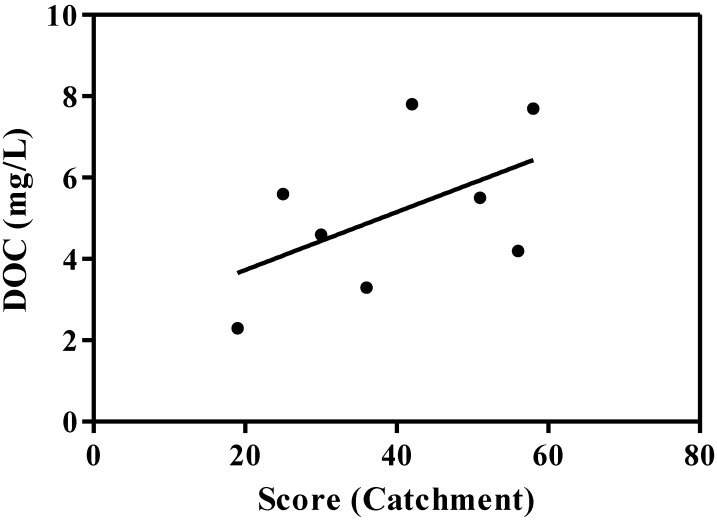
Relationship between score (catchment characteristics questionnaire) and DOC (n = 8, R^2^ = 0.28).

Firstly, the risk analysis was conducted on the eight test catchments and compared with mean DOC concentrations obtained from source waters data released by environmental and sanitary monitoring networks between 1993 and 2011. The DOC parameter was chosen as it could be considered as a proxy of raw sources water quality (Figure 7). While there is no significant linear correlation, higher DOC concentrations are generally associated with higher risk scores.

Secondly, for the purpose of validating the assessment of risk posed by selected substances, the data were compared with field data collected by the environment and health authorities. The majority of substances selected based on existing pressures on the catchment were detected in source waters. Moreover, the substances that are not selected by the DSS are effectively not present in raw waters, proving that the contaminant selection procedure does not omit potentially harmful contaminants (Table 2).

**Table 2:** Comparison between contaminants selected by the DSS and those currently detected in waters.

Sampling Site	Number of Contaminants Selected by the DSS and Analyzed *	Contaminants Detected * (%)	Number of Contaminants Non Selected by the DSS and Analyzed *	Contaminants Detected * (%)
1	11	45.5	0	-
2	12	91.7	0	-
3	12	66.7	0	-
4	0	-	20	0.0
5	12	91.7	7	0.0
6	0	-	20	10.0
7	12	16.7	14	0.0
8	11	9.1	12	0.0
9	14	100	6	0.0

Note: * during the field sampling campaigns.

## 4. Discussion

By assessing the vulnerability of a water system using a semi-quantitative approach combined with substances identification and health risk assessment, our decision support system identifies potential problems and attests to the systems suitability for assessing the status of water quality. Moreover, the statistically-based DOC model provides a useful guide to key factors and processes determining DOC loadings to water treatment plants draining natural upland catchments. Future iterations should enable responses to future environmental change to be assessed, thus providing significant economic benefits to the water industry by informing planning procedures.

A number of issues require further development and testing before the DSS is suitable for widespread use by water treatment managers. First, the scoring system used to rate individual criteria stipulated in the catchment questionnaire and drawn largely from the existing literature requires more methodical ground-truthing to ensure that appropriate weighting has been applied.

Second, while the statistical modeling of spatial variation in DOC for natural environments appears to indicate significant roles for both temperature and precipitation, it is still unclear to what extent these factors will influence trends at individual sites over time. Further work is ongoing to determine their importance, and quantification of these effects will be necessary before a dynamic climate change element can be incorporated.

Third, the calculation of contaminant concentrations in treated waters is based on removal rates which are determined following a literature review of existing knowledge, some values then could be modified in the near future. Moreover, this calculation is also based on environmental concentrations available in the literature and could not reflect the existing background concentrations for a given catchment.

Fourth, the DSS is limited to specific substances for which toxicological data exist. An improvement in knowledge for emerging substances is needed to complete the database, such as for instance, data for antibiotics and anticancer drugs. The DSS will be improved as new toxicological information will be released.

Finally, the physical structure of the water distribution network is likely to exert a significant influence on variation in water quality. Residence times and material contact could lead to an increase of disinfection by-products formation potential, especially for high water temperatures. It has been shown that THM concentrations could significantly vary from 2–4 times within the distribution system [70]. The residence time could also play a role on biofilm and microorganism’s development [71]. Furthermore, the material from which water pipes are constructed should be considered with respect to the potential to the release of endocrine disrupting substances such as organotins [72] and carcinogenic substances such as vinyl chloride monomer [73].

## 5. Conclusions

The issue of drinking water quality compliance in small and medium scale water services is of paramount importance in relation to the requirements of the DWD and the existing difficulties in its implementation within such distribution systems. Various incentives such as free training, advice and tools play a key role for successful DWD implementation in SSWS. In order to help water authorities and managers tackle issues regarding the microbiological, chemical and climate risks in their water treatment plants, a decision support system has been defined and tested on different catchments representing a wide range of environments. ARTEM-WQ has been developed using a sequential approach based on a risk analysis in conjunction with catchment characteristics and treatment operations. The DSS allows operators to evaluate information on the current global status of the water system, while also assessing human health risks of substances potentially present in finished waters. In combination, this information allows formulation of recommendations for improvement while supporting decision making in its widest context. Initial testing of the tool in various catchments shows promising potential to inform water managers of risks and appropriate mitigative actions. Further improvements are now needed, however, including advancement of toxicological knowledge, environmental background pollutant concentrations and their respective removal rates. Moreover, the impact of distribution systems on water quality variation should also be included in the next version of the DSS.

## Supplementary Files

Supplementary File 1 Supplementary Information (PDF, 359 KB)
