# Supplementary material for: A Decision Support System for Drinking Water Production Integrating Health Risks Assessment
Source: Int J Environ Res Public Health. 2014 Jul 18;11(7):7354–75. doi: 10.3390/ijerph110707354 (PMC4113881; doi:10.3390/ijerph110707354)
Supplement: Supplementary File 1 — Supplementary Information (PDF, 359 KB) [file ijerph-11-07354-s001.pdf]

A Decision Support System for Drinking Water Production Integrating Health Risks Assessment

Table S1. Anthropized catchment Risk Assessment.

Item	Factor	Choice	Risk Score
**Animals**	Cattle/calves livestock unit per ha of forage area (LDI *****)	Absence	0
		≤1	1
		>1	3
	Sheep/lambs livestock unit per ha of forage area (LDI *****)	Absence	0
		≤1	1
		>1	3
	Pig livestock unit per ha of forage area (LDI *****)	Absence	0
		≤1	1
		>1	3
	Poultry livestock unit per ha of forage area (LDI *****)	Absence	0
		≤1	1
		>1	3
	Any other farmed animals or birds	Absence	0
		Presence	1
	Evidence of wildlife around the catchment	Absence	0
		Presence	2
	Animals access to water sources including feeder streams	No access (natural or artificial barrier)	0
		Access possible at a distance > 200 m from the catchment	1
		Access possible at a distance < 200 m from the catchment	3
**Agricultural practices within the catchment**	Farm waste (Slurry/Dung) or sewage sludge spreading within the catchment	Absence	0
		Presence	3
	Slurry/dung open stores	Absence	0
		Presence	3
	Cultivated lands with crops	Absence	0
		<20%	1
		20%–50%	2
		>50%	3

Int. J. Environ. Res. Public Health **2014**, *11*

Table S1. *Cont.*

Item	Factor	Choice	Risk Score
**Agricultural practices within the catchment**	Cultivated lands with orchards/vineyards	Absence	0
		<10%	1
		10%–30%	2
		>30%	3
	Awareness of the presence of drinking water supply/sources by agricultural workers	Good awareness	0
		No Awareness	2
	Full compliance with Good Agricultural Practice Regulations verified by catchment inspection	Yes	0
		No	3
**Urban & Industrial Discharge**	Septic tanks serving population	Absence	0
		Presence	2
	Flooding of septic tanks on flood plains	Yes	2
		No	0
	Pop equivalent served by all wastewater Works	Absence	0
		<5000	1
		5000–50,000	2
		>50,000	3
	Presence of tourism infrastructures served by private on-site wastewater treatment systems	Yes	2
		No	0
	Presence of hospital	Yes	3
		No	0
	Presence of industries	Yes	3
		No	0
	Evidence of malfunction on one or more on-site wastewater treatment systems	Yes	3
		No	0
	Presence of landfill sites in the catchment	Yes	3
		No	0
	Wastewater works discharge distance from catchment point	Absence	0
		>10 km	1
		≤10 km	2

Int. J. Environ. Res. Public Health **2014**, *11*

Table S1. *Cont.*

Item	Factor	Choice	Risk Score
**Urban & Industrial Discharge**	Storm water overflows	Absence	0
		Presence	3
	Integrated Pollution management plan discharge from intensive agricultural activity or agricultural related discharge	Yes	−1
		No	2
	All wastewater treatment plants complying with the Urban WasteWater Treatment Directive quality standards	Yes	0
		No	2
	UV inactivation at outlet of wastewater treatment plants	Yes	−1
		No	1
**Catchment characteristics**	Water source type	Upland reservoir/lake	1
		Lowland long term storage reservoir/lake	2
		Upland river or stream—bank side storage	2
		Upland river or stream—direct abstraction	3
		Lowland river or stream—direct abstraction or bank side storage	3
	Slopes	>10% near the catchment	2
		≤10% near the catchment	0
	DOC Trends	Decreasing	−2
		Stable	0
		Increasing	2
**Catchment management**	Catchment inspections frequency	1 per month or more	−1
		Between monthly and annually	0
		1 per year or less	1
	Procedures in place to deal with irregularities on the catchment	In place	−1
		No procedure	1
	Catchment protection areas implementation	In place	−1
		Procedure started	0
		Procedure not started	1

Note: * LDI: Livestock Density Index

Int. J. Environ. Res. Public Health **2014**, *11*

Table S2. Natural catchment Risk Assessment.

Item	Factor	Choice	Risk Score
**Animals**	Cattle/calves livestock unit per ha of forage area (LDI *****)	Absence	0
		≤1	1
		>1	3
	Sheep/lambs livestock unit per ha of forage area (LDI *****)	Absence	0
		≤1	1
		>1	3
	Pig livestock unit per ha of forage area (LDI *****)	Absence	0
		≤1	1
		>1	3
	Poultry livestock unit per ha of forage area (LDI *****)	Absence	0
		≤1	1
		>1	3
	Any other farmed animals or birds	Absence	0
		Presence	1
	Evidence of wildlife around the catchment	Absence	0
		Presence	2
	Animals access to water sources including feeder streams	No access (natural or artificial barrier)	0
		Access possible at a distance > 200 m from the catchment	1
		Access possible at a distance < 200 m from the catchment	3
**Agricultural practices within the catchment**	Farm waste (Slurry/Dung) or sewage sludge spreading within the catchment	Absence	0
		Presence	3
	Slurry/dung open stores	Absence	0
		Presence	3
	Awareness of the presence of drinking water supply/sources by agricultural workers	Good awareness	0
		No Awareness	2
	Full compliance with Good Agricultural Practice Regulations verified by catchment inspection	Yes	0
		No	3

Table S2. *Cont.*

Item	Factor	Choice	Risk Score
**Urban & Industrial Discharge**	Septic tanks serving population	Absence	0
		Presence	2
	Flooding of septic tanks on flood plains	Yes	2
		No	0
	Presence of Wastewater Works	Yes	1
		No	0
	Presence of tourism infrastructures served by private on-site wastewater treatment systems	Yes	2
		No	0
	Presence of hospital	Yes	3
		No	0
	Presence of industries	Yes	3
		No	0
	Evidence of malfunction on one or more on-site wastewater treatment systems	Yes	3
		No	0
	Presence of landfill sites in the catchment	Yes	3
		No	0
	Wastewater works discharge distance from catchment point (how much PE?)	Absence	0
		>10 km	1
		≤10 km	2
	Storm water overflows	Absence	0
		Presence	3
	Integrated Pollution management plan discharge from intensive agricultural activity or agricultural related discharge	Non applicable	0
		Yes	−1
		No	2
	All wastewater treatment plants complying with the Urban WasteWater Treatment Directive quality standards	Non applicable	0
		Yes	0
		No	2
	UV inactivation at outlet of wastewater treatment plants	Non applicable	0
		Yes	−1
		No	1

Int. J. Environ. Res. Public Health **2014**, *11*

Table S2. *Cont.*

Item	Factor	Choice	Risk Score
**Catchment characteristics**	Water source type	Upland reservoir/lake	1
		Lowland long term storage reservoir/lake	2
		Upland river or stream—bank side storage	2
		Upland river or stream—direct abstraction	3
		Lowland river or stream—direct abstraction or bank side storage	3
	Slopes	>10% near the catchment	2
		≤10% near the catchment	0
	DOC Trends	Decreasing	−2
		Stable	0
		Increasing	2
**Catchment management**	Catchment inspections frequency	1 per month or more	−1
		Between monthly and annually	0
		1 per year or less	1
	Procedures in place to deal with irregularities on the catchment	In place	−1
		No procedure	1
	Catchment protection areas implementation	In place	−1
		Procedure started	0
		Procedure not started	1

Note: ***** LDI: Livestock Density Index

Int. J. Environ. Res. Public Health **2014**, *11*

Table S3. Treatment Risk Assessment.

Item	Factor	Choice	Risk Score
**Water treatment processes**	Water treatment processes	Buffer tank	−1
		Simple sand filtration (not slow sand filtration)	−1
		Coagulation with aluminium	−2
		Coagulation with ferric chloride	−2
		DAF/sedimentation and filtration	−1
		Rapid gravity or pressure filtration	−2
		Slow sand filtration	−2
		Granular activated carbon	−2
		Powder activated carbon	−2
		Membrane filtration (approved)	−3
		Membrane filtration (not approved)	−2
		Chlorination (HClO, ClO2, Cl2 gazeous)	−2
		UV disinfection	−2
		Nanofiltration	−2
		Ozonation	−2
**Raw water intake management**	Water quality monitor	Not appropriate	1
		Appropriate alarmed and connected to telemetry	−1
	Intake shut down when poor water quality	No	1
		Manual	−1
		Automatic	−2
**Treatment works monitoring**	Coagulation : coagulant dose (and or pH) control	Absence	2
		Manual control (not flow proportional)	0
		Monitored and alarmed	−1
	After clarification/Filtration—water turbidity meter/ particle counter	No turbidity meter or particle count	1
		turbidity meter/particle count but no alarm on telemetry	0
		turbidity meter/particle count with alarm on telemetry	−1

Table S3. *Cont.*

Item	Factor	Choice	Risk Score
**Treatment works monitoring**	After clarification/Filtration—monitoring for residual coagulant	Not monitored	0
		Routine discrete monitoring for residual coagulant	−1
		Continuous monitoring for residual	−2
	Membrane filtration—Plant monitored for integrity	Non applicable	0
		Not monitored	1
		Plant monitored for integrity but not alarmed	0
		Plant monitored and alarmed for integrity	−1
	Membrane filtration—Particle counter used continuously to monitor filter performance	Non applicable	0
		Yes	−1
		No	1
	Disinfection—Plant monitored for integrity and correct dosage	Not monitored	1
		Plant monitored for integrity and correct dosage but no alarm	0
		Plant monitored and alarmed for integrity and correct dosage	−1
**Filter performance**	Treated water turbidity increases range, excluding normal backwash period or turbidity in the final water >1.0 NTU	Less than 50%	0
		More than 50%	1
**Disinfection performance**	UV inactivation—Influent turbidity consistently	<0.2 NTU	−2
		0.2–1.0 NTU	−2
		>1.0 NTU	−1
	Free chlorine residual consistently	Not monitored or <0.1 mg/L	2
		0.1–0.3 mg/L	−2
		>0.3 mg/L	1
**Treatment works operation**	Plant with documented management systems that includes procedures and process maintenance/control manuals	Yes—complete	−1
		Yes but incomplete	0
		No	1
	Process control manuals specific to works available	Yes—complete	−1
		Yes but incomplete	0
		No	1

Table S3. *Cont.*

Item	Factor	Choice	Risk Score
**Treatment works operation**	Auditable action plans available for dealing with deviations in quality and evidence of implementation of the plan	Available—complete	−1
		Available but incomplete	0
		Not available	1
	WTP inspections carried out	>1 per month or more	−1
		Between monthly and annually	0
		1 per year or less	1
	Water flow through works when operating has increased by >10% in <30 min in last 12 months	Yes	1
		No	0
	Flow through works above design flow for >10% of time in last 12 months	Yes	2
		No	0
	Flow through works >130% above design flow for >50% of time in last 12 months	Yes	2
		No	0
	Filters bypassed during the year	Yes	1
		No	0

© 2014 by the authors; licensee MDPI, Basel, Switzerland. This article is an open access article distributed under the terms and conditions of the Creative Commons Attribution license (http://creativecommons.org/licenses/by/3.0/).
